# MicroRNA-205 targets SMAD4 in non-small cell lung cancer and promotes lung cancer cell growth *in vitro* and *in vivo*

**DOI:** 10.18632/oncotarget.10339

**Published:** 2016-06-30

**Authors:** Yuanyuan Zeng, Jianjie Zhu, Dan Shen, Hualong Qin, Zhe Lei, Wei Li, Zeyi Liu, Jian-an Huang

**Affiliations:** ^1^ Department of Respiratory Medicine, The First Affiliated Hospital of Soochow University, Suzhou, P. R. China; ^2^ Institute of Respiratory Diseases, Soochow University, Suzhou, P. R. China; ^3^ Department of Cardiothoracic Surgery, The First Affiliated Hospital of Soochow University, Suzhou, P. R. China; ^4^ Suzhou Key Laboratory for Molecular Cancer Genetics, Suzhou, P. R. China; ^5^ Department of Oncology, The First Affiliated Hospital of Soochow University, Suzhou, P. R. China

**Keywords:** SMAD4, miR-205, NSCLC, proliferation, cell cycle

## Abstract

Despite advances in diagnosis and treatment, the survival of non-small cell lung cancer (NSCLC) patients remains poor; therefore, improved understanding of the disease mechanism and novel treatment strategies are needed. Downregulation of SMAD4 and dysregulated expression of miR-205 have been reported. However, the relationship between them remains unclear. We investigated the effect of microRNA (miR)-205 on the expression of *SMAD4* in NSCLC. Knockdown and overexpression of *SMAD4* promoted or suppressed cellular viability and proliferation, and accelerated or inhibited the cell cycle in NSCLC cells, respectively. The 3′-untranslated region (3′-UTR) of *SMAD4* was predicted as a target of miR-205. Luciferase assays validated that miR-205 binds directly to the *SMAD4* 3′-UTR. Protein and mRNA expression analyses confirmed that miR-205 overexpression in NSCLC cells inhibited the expression of SMAD4 mRNA and protein. In human NSCLC tissues, increased miR-205 expression was observed frequently and was inversely correlated with decreased *SMAD4* expression. Ectopic expression of miR-205 in NSCLC cells suppressed cellular viability and proliferation, accelerated the cell cycle, and promoted tumor growth of lung carcinoma xenografts in nude mice. Our study showed that miR-205 decreased *SMAD4* expression, thus promoting NSCLC cell growth. Our findings highlighted the therapeutic potential of targeting miR-205 in NSCLC treatment.

## INTRODUCTION

Worldwide, lung cancer is a primary cause of cancer-related death [1], and among lung cancer patients, more than 80 percent have non-small cell lung cancer (NSCLC). Despite improvements in cancer treatment, the 5-year survival rate is currently only 15%. Early stage diagnosis and surgery of NSCLC pateints could result in a 5-year survival rate of up to 55–80% [2]. Thus, a better understanding of the mechanisms of NSCLC development and progression are important for early diagnosis and prevention, as well as targeted treatment.

MicroRNAs (miRNAs) are non-coding RNA molecules of approximately 19–24 nucleotides in length that repress the translation or promote the degradation of target mRNAs [3, 4]. It has been estimated that miRNAS regulate upto 30% of mRNAs [5]. MiRNAs have important effects on diverse biological and pathological processes, including tumor cell proliferation, differentiation, and survival [6–8]. The expressions of miRNAs appear to be tissue or tumor type-specific, specific miRNA expression signatures or panels could even classify human cancers [9], distinguish tumor subtypes [10], and correlate with prognosis [11]. MiR-205, which is located in a lung cancer-associated genomic amplification region at 1q32.2. Dysregulation of miR-205 was observed in many types of tumors, including lung cancer [12]. Recently, it was demonstrated that loss of miR-205 promoted the epithelial to mesenchymal transition (EMT) during tumor progression [13]. In addition, another study showed that low miR-205 expression in mammary epithelial cells promoted EMT, while its overexpression repressed cancer cells stemness [14]. Moreover, the expression of miR-205 was higher in squamous cell lung carcinoma compared with other types of NSCLC [10].

As a Co-Smad of the Smad family, Smad4 was identified as a tumor suppressor gene. It is a common mediator of transforming growth factor-β (TGF-β) signaling and is involved in TGF-β-induced growth inhibition [15]. SMAD4-dependent TGF-β signaling is common during tumor development and progression; can inhibit cell proliferation, promote cell motility and the EMT process in most epithelial cells; and affects sensitivity to clinical therapy [16–18]. Inactivation of SMAD4-induced deregulation of the TGF-β superfamily signaling is well established in some cancers. Moreover, SMAD4 was associated with tumor invasion, metastasis and prognosis in different cancers [19, 20]. Although the function of SMAD4 is important, it remains unclear how SMAD4 is regulated at the transcriptional level in human NSCLC, especialy with respect to the involvement of miRNAs.

In the present study, we examined the role of miR-205 and SMAD4 in NSCLC together with its clinical characteristics and cell phenotypes. The primary tumors and adjacent normal tissues in a cohort of 52 patients were analyzed by quantitative real-time reverse transcription polymerase chain reaction (qRT-PCR) for the expression of miR-205 and *SMAD4*. The functions of miR-205 and its molecular link to SMAD4 were also investigated in cell lines and transplanted tumor models of lung carcinoma in mice. Our results indicated the importance of miR-205 as a potential target in clinical therapy and demonstated that this miRNA merits further investigation as a promising gene therapy target to treat NSCLC.

## RESULTS

### The expression SMAD4 is decreased in NSCLC tissues and cell lines

**Figure 1:**
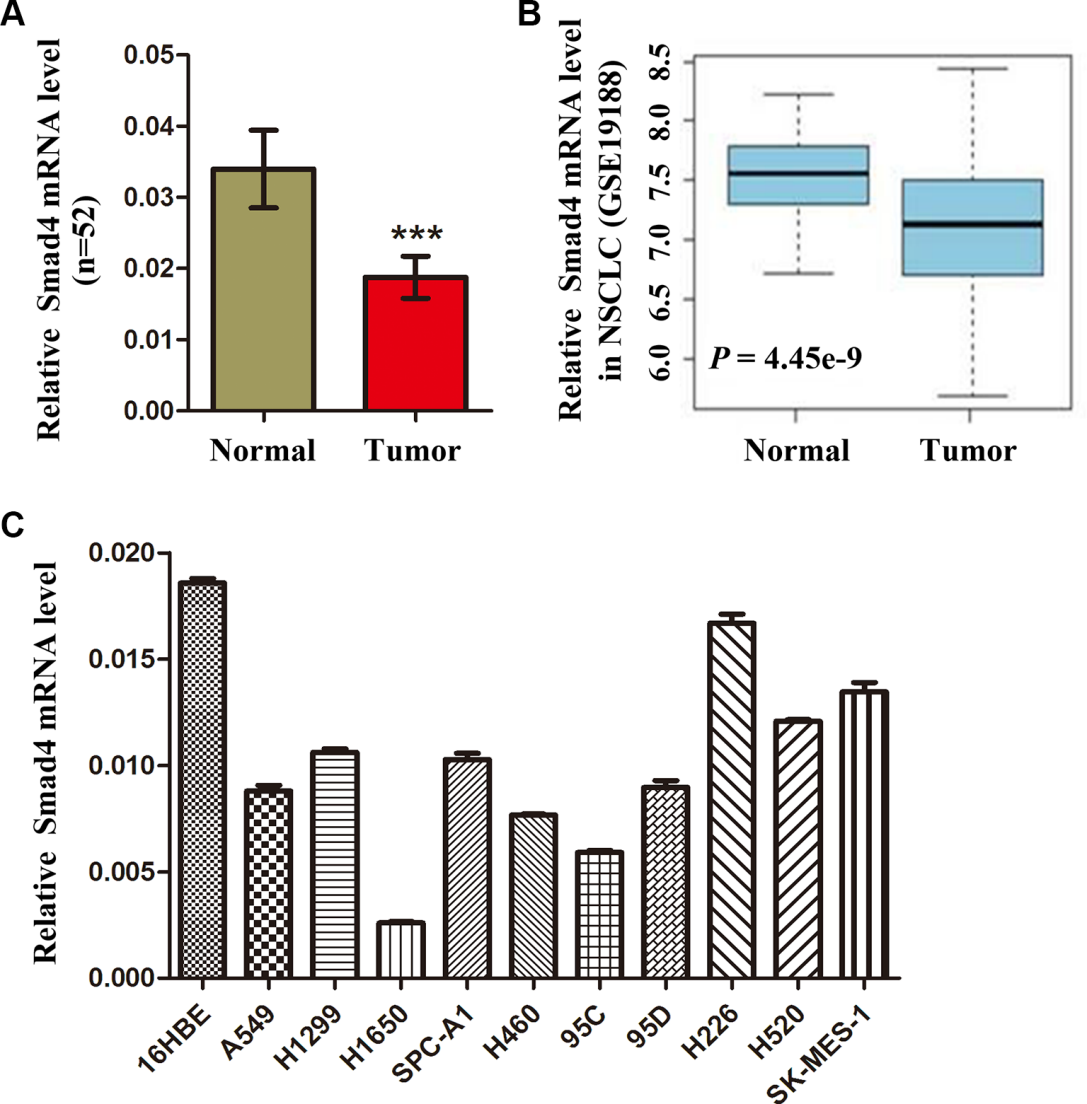
Expression of SMAD4 is reduced in NSCLC cells and human NSCLC tissues (**A**) *SMAD4* mRNA levels in 52 NSCLC tissues and paired noncancerous lung tissues. (**B**) Box plots showing relative *SMAD4* mRNA expression levels of NSCLC tumors and adjacent normal lung tissues in a public data set (GSE19188). (**C**) Quantitative real-time reverse transcription PCR analysis of *SMAD4* mRNA levels in HBE cells and NSCLC cells (A549, H1299, A1650, SPC-A1, H460, 95d, 95C, H226, H520 and SK-MES-1). *SMAD4* mRNA levels are expressed as a relative index normalized against the expression of *ACTB* (β-actin). *P < 0.05; **P < 0.01; ***P < 0.001.

SMAD4, a tumor suppressor, is frequently reduced in cancer tissue and is associated with evolving neoplasms. To determine whether SMAD4 expression is reduced in NSCLC, we detected *SMAD4* mRNA expression in 52 paired NSCLC tissues and adjacent noncancerous normal tissues. The results showed that *SMAD4* mRNA levels were significantly lower in NSCLC tissues than in adjacent noncancerous lung tissues (Figure 1A). Furthermore, a public data set (GSE19188) showed that the expression of *SMAD4* mRNA was downregulated in human NSCLC tissues (Figure 1B). To determine the function of *SMAD4* expression during NSCLC development and progression, we correlated *SMAD4* expression with clinicopathological characteristics in NSCLC patients, including gender, age, histological type, TNM staging, smoking history and differentiation. We found higher *SMAD4* expression in adenocarcinomas compared with other types of NSCLC (*P* = 0.02). Interestingly, we also observed lower expression of miR-205 in adenocarcinomas than in squamous cell lung carcinoma (Table 1). Furthermore, we detected *SMAD4* mRNA expression in 10 NSCLC cell lines: *SMAD4* mRNA levels were significantly lower in NSCLC cell lines than in HBE cells (Figure 1C).

**Table 1:** Clinical characteristics and levels of miR-205 and *Smad4* mRNA expression in NSCLC tissues

Characteristics	*n* (%)	miR-205 expression	*Smad4* mRNA expression
Age			
≤ 65	23 (44.2%)	0.04636 ± 0.03018	0.01468 ± 0.005275
> 65	29 (55.8%)	0.02214 ± 0.005835	0.01613 ± 0.002599
*P* value		0.2542	0.3176
Gender			
Male	35 (67.3%)	0.03881 ± 0.02009	0.01737 ± 0.003710
Female	17 (32.7%)	0.007869 ± 0.004414	0.02170 ± 0.004920
*P* value		0.2933	0.497
Histology			
Adenocarcinomas	23 (44.2%)	0.002255 ± 0.001046	0.02318 ± 0.004031
Squamous cell carcinomas	21 (40.4%)	0.06717 ± 0.03249	0.01657 ± 0.005662
Others	8 (15.4%)	0.003701 ± 0.002517	0.01197 ± 0.003196
*P* value		0.0002	0.0118
Smoking status			
Yes	29 (55.8%)	0.04599 ± 0.02409	0.01802 ± 0.004432
No	23 (44.2%)	0.006882 ± 0.003348	0.01976 ± 0.003768
*P* value		0.1577	0.7734
Clinical stage			
I	14 (26.9%)	0.02205 ± 0.01011	0.01707 ± 0.004104
II	11 (21.2%)	0.005553 ± 0.003258	0.01591 ± 0.002582
III	21 (40.4%)	0.01759 ± 0.008529	0.02082 ± 0.006296
IV	6 (11.5%)	0.1255 ± 0.1127	0.02095 ± 0.009091
*P* value		0.7945	0.7752

Data are presented as mean ± SE. An unpaired *t* test was used for two groups. The Kruskal–Wallis test was used for three or more groups.

### The function of SMAD4 in NSCLC cells

**Figure 2:**
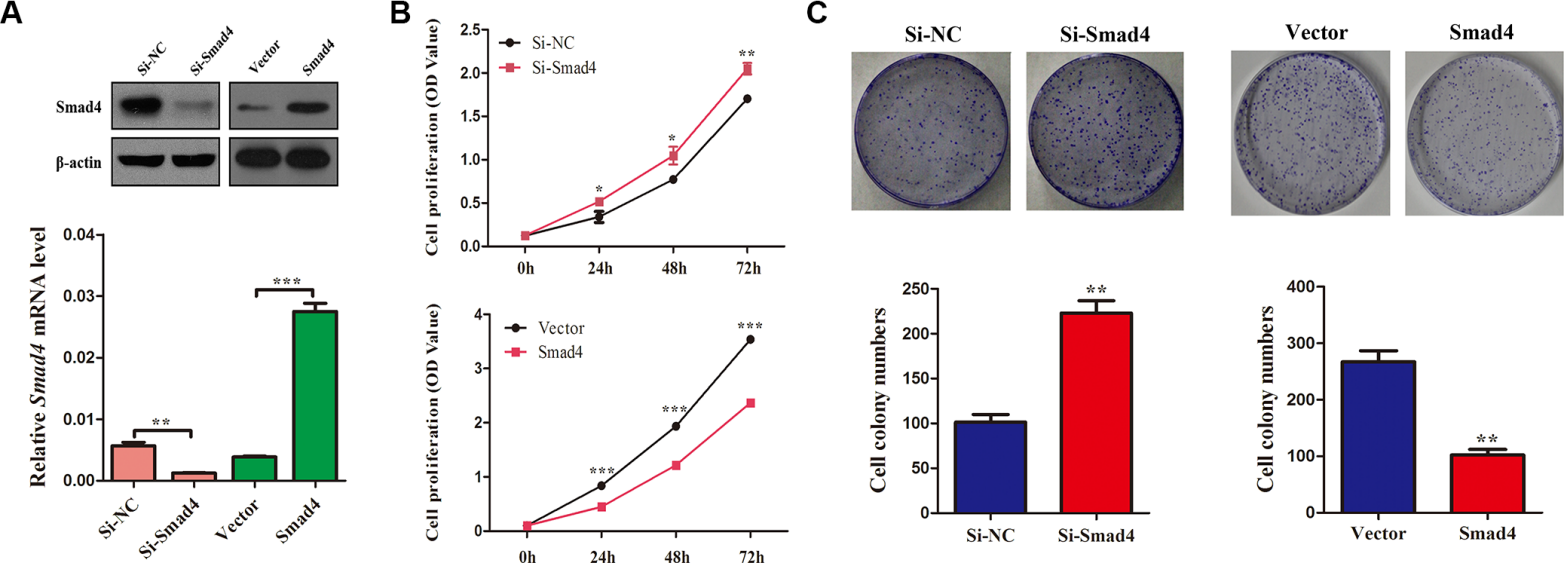
Silencing of *SMAD4* promotes NSCLC cell viability and proliferation and overexpression *SMAD4* inhibits NSCLC cell viability and proliferation (**A**) SMAD4 mRNA and protein levels in A549 cell lines either silenced for *SMAD4* expression or overexpressing *SMAD4*, respectively. (**B**) CCK-8 assay of cell viability in A549 cells; the results were detected at 24, 48 and 72 h. (**C**) Representative images of clonogenic analysis for cell proliferation in A549 cells. Bar charts showing clonogenic growth of A549 cells. Values are the means ± SE from three measurements. *P < 0.05; **P < 0.01; ***P < 0.001.

Considering the hypothesis that loss of SMAD4 inhibits cell proliferation, firstly, we used a specific siRNA targeted against *SMAD4* (si-Smad4) to reduce the expression of *SMAD4* in NSCLC cells. In addition, stable A549 cell lines overexpressing *SMAD4* were generated. The successful knockdown and overexpression of *SMAD4* were confirmed by qRT-PCR and western blotting (Figure 2A), Cell growth was promoted significantly in cells transfected with si-Smad4 compared with the control cells. By contrast, in the stable cell lines overexpressing Smad4, cell growth was significantly suppressed compared with the control cells, at 24 h, 48 h, 72 h after transfection (Figure 2B). Furthermore, to validate these results, we used a clonogenic assay to detect cell growth, and observed similar results (Figure 2C).

### Knockdown of *SMAD4* promotes, and *SMAD4* overexpression inhibits, the cell cycle in NSCLC cells

**Figure 3:**
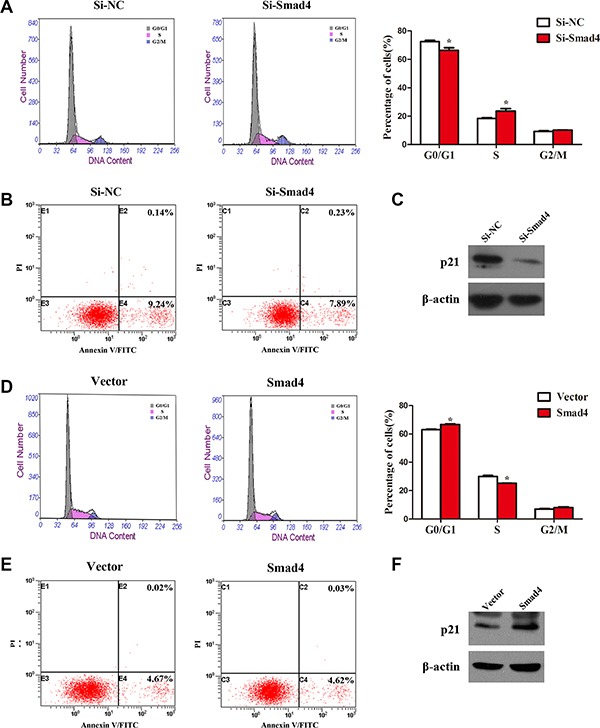
Knockdown of *SMAD4* or overexpression of SMAD4 had no effect on cell apoptosis, whereas it promotes or inhibits the cell cycle in NSCLC cells (**A**) and (**D**) Flow cytometry cell cycle analysis of A549 cells (silenced for *SMAD4* or overexpressing *SMAD4* and compared with NC or Vector controls). Cells were harvested at 72 h post-transfection and stained with propidium iodide. The percentages of cell cycle phases are shown in the insets of each panel, in which values represent mean ± SD of three measurements. (**B**) and (**E**) Flow cytometry apoptosis assay of A549 cells (silenced for *SMAD4* or overexpressing *SMAD4* and compared with NC or Vector controls). Cells were harvested at 72 h post-transfection and stained with Annexin V/FITC and propidium iodide (PI). (**C**) and (**F**) Expression of p21 (an inhibitor of cell proliferation) was analyzed by western blotting. Densitometry values for each protein were normalized to β-actin. *P < 0.05; **P < 0.01; ***P < 0.001.

To further investigate how SMAD4 affects NSCLC cell growth, we examined cell apoptosis and distribution of cell cycle phases in *SMAD4*-silenced and *SMAD4*-overexpressing A549 cell lines. Transfection with si-Smad4 or its overexpression in A549 cells had no effect on cell apoptosis (Figure 3B and 3E), whereas we observed significant changes in the proportion of cells in the G1 and S phases (Figure 3A and 3D). Knockdown of *SMAD4* caused a decrease in the number of cells in the G0/G1 phase and an increase in the S phase. By contrast, overexpression of *SMAD4* caused accumulation of cells in the G0/G1 phase and reduced levels in the S phase. To further validate our results, we detected the expression of p21, which inhibits cell growth [21]: knockdown of *SMAD4* repressed the expression of p21, while overexpression of *SMAD4* enhanced p21 expression (Figure 3C and 3F). Collectively, the results suggested that SMAD4 inhibits cell proliferation in NSCLC via the cell cycle.

### SMAD4 expression is regulated by miR-205 through targeting its 3′-UTR in NSCLC

**Figure 4:**
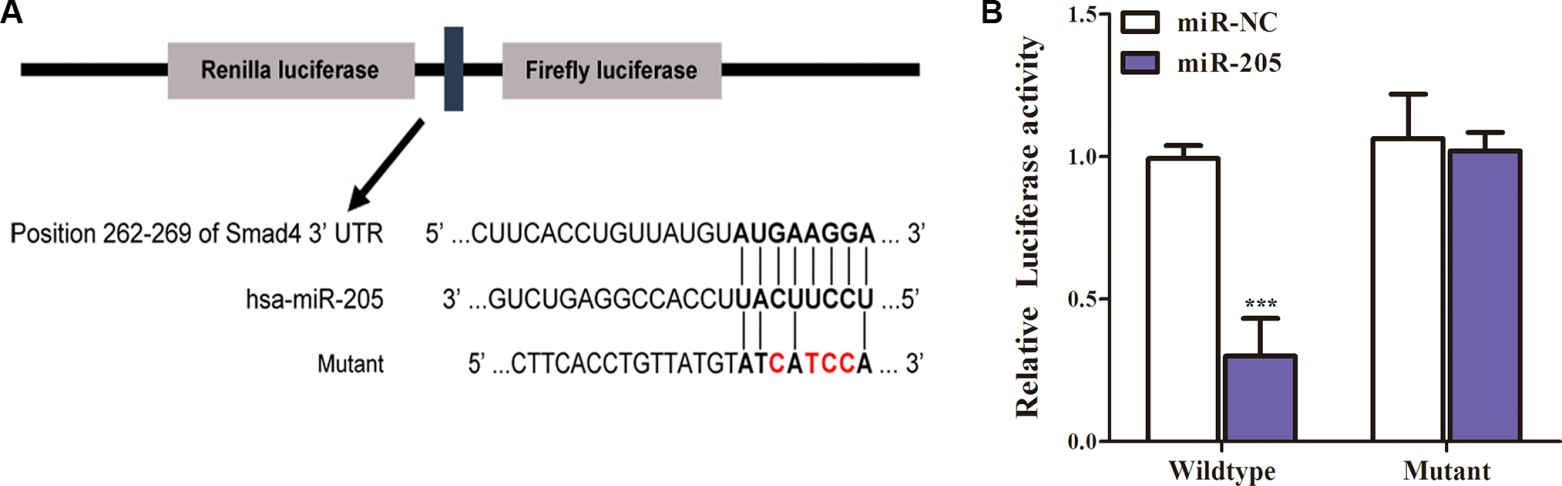
MiR-205 reduces *SMAD4* expression by directly targeting its 3 ′**-UTR**. (**A**) Schematic diagram showing the subcloning of the predicted miR-205 binding site at position 262–269 of the *SMAD4* 3′-UTR into a psiCHECK-2 luciferase construct. Predicted duplex formation between miR-205 and the wild-type or mutant of miR-205 binding site is indicated. (**B**) Luciferase activities of the construct containing the wild-type or mutant Smad4 3′-UTR reporter gene in A549 cells cotransfected with negative control (NC) or miR-205. Scrambled sequences were used as the NC. Relative Renilla luciferase activity was determined followed by normalization against the firefly luciferase activity.

MiRNAs can inhibit or suppress various biological processes including cell proliferation by targeting proliferation-related genes [22], and Huang *et al*. [23] showed that *in silico, SMAD4* was a target gene of miR-205; therefore, we hypothesized that miR-205 could inhibit *SMAD4* expression by binding to the *SMAD4* 3′-UTR region. To test this possibility, we subcloned the *SMAD4* 3′-UTR, containing the putative miR-205 binding site (both the wild type and mutated sites, separately) into vector psiCHECK-2 (Figure 4A). MiR-205 is significantly downregulated in NSCLC cell lines [24, 25]; therefore, we only transiently cotransfected the reporter construct with miR-205 mimics into A549 cells. The results showed that the miR-205 mimics significantly inhibited the luciferase activity in cells transfected with the wild-type *SMAD4* 3′-UTR but did not repress the luciferase activity in cells containing the mutant construct (Figure 4B). Moreover, overexpression of miR-205 reduced *SMAD4* expression in NSCLC cells remarkably (Figure 6A). Taken together, the results suggested that miR-205 binds directly to the target site the 3′-UTR of *SMAD4* in NSCLC cells to inhibit its expression.

**Figure 5:**
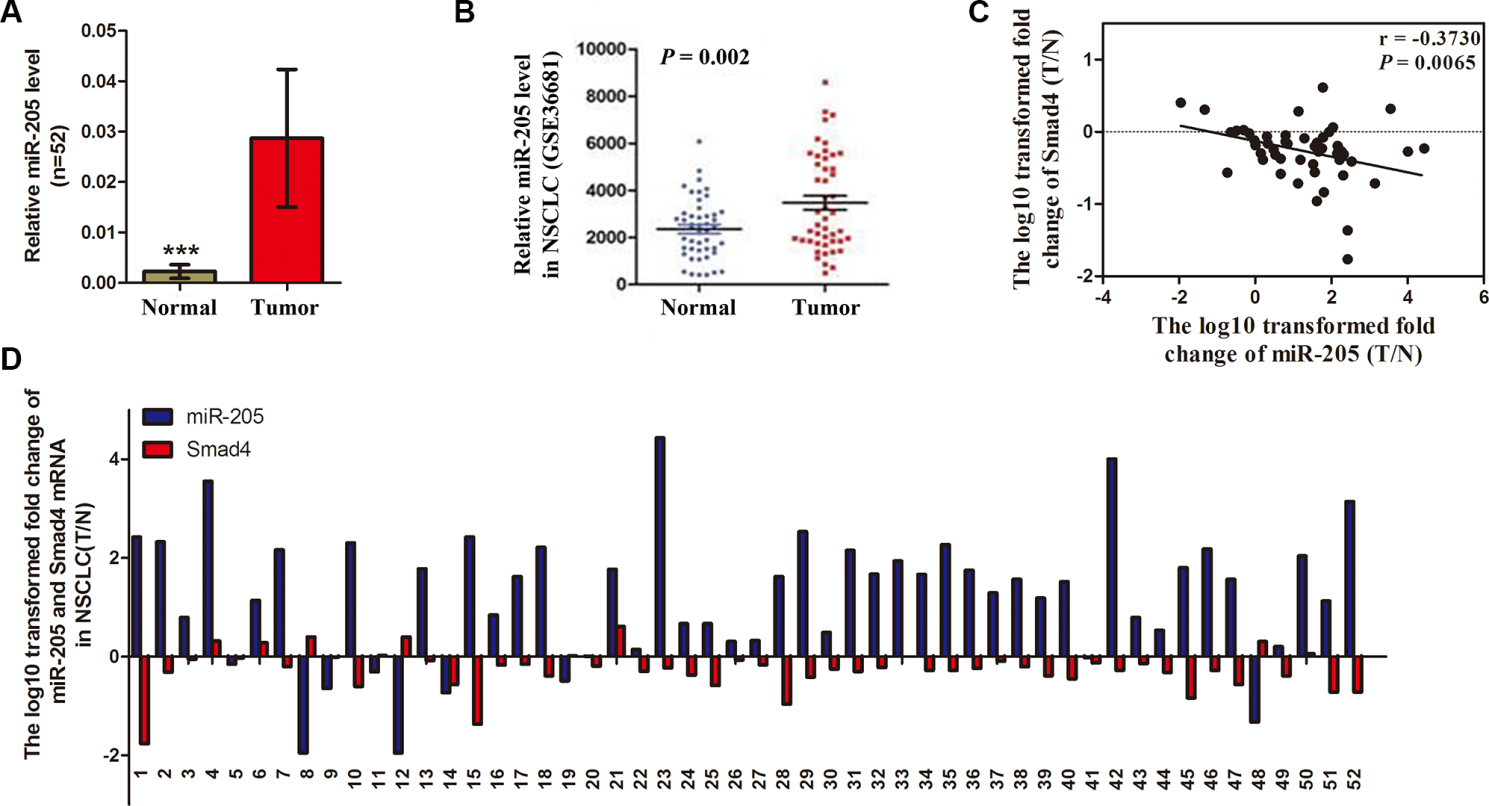
Expression of miR-205 is upregulated in NSCLC tissues and inversely correlated with *SMAD4* expression (**A**) Relative miR-205 levels in 52 NSCLC tissues (T) and paired noncancerous lung tissues (N). (**B**) Scatter diagram showing relative *SMAD4* mRNA expression levels of NSCLC tumors and adjacent normal lung tissues in a public data set (GSE36681). (**C**) Correlation between the miR-205 level and *SMAD4* mRNA expression in 52 paired NSCLC tissues. MiR-205 and *SMAD4* mRNA levels are expressed as relative indexes normalized against U6 and β-actin, respectively. The x and y-axes represent the log10 transformed fold change of T/N mRNA expression ratios of miR-205 and *SMAD4*, respectively. (**D**) Relative expression of miR-205 levels and *SMAD4* mRNA in 52 paired NSCLC tissues. The y-axis represents the log10 transformed fold change of T/N expression ratios of miR-205 levels and SMAD4 mRNA. The number of each specimen is indicated below the x-axis. *P < 0.05; **P < 0.01; ***P < 0.001.

**Figure 6:**
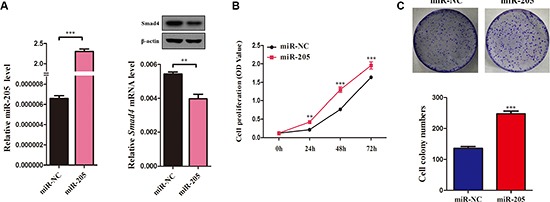
Overexpression of an miR-205 mimic inhibits NSCLC cell viability and proliferation (**A**) QRT-PCR analysis of miR-205 levels in A549 cells transfected with miR-205 mimics or NC for 72 h. U6 was used as the internal control. *SMAD4* mRNA expression in A549 cells transfected with miR-205 mimics or NC. β-actin was used as the internal control. (**C**) The SMAD4 protein levels in A549 cells transfected with miR-205 mimics or NC. (**B**) CCK-8 assay of cell viability in A549 cells transfected with miR-205 mimics or NC for 96 h. B Representative images of the clonogenic analysis for cell proliferation in A549 cells transfected with miR-205 mimics or NC. Bar charts showing clonogenic growth of A549 cells transfected with miR-205 mimics or NC. Values are represented as means ± SE from three measurements. **P < 0.01; ***P < 0.001.

### The miR-205 level is increased in NSCLC tissues and ectopic miR-205 expression can promote NSCLC cell proliferation

As illustrated in Figure 5A, among 52 randomly selected paired tissues from NSCLC patients, miR-205 expression was significantly increased in tumor tissues compared with paired noncancerous tissues. Furthermore, Gene Expression Omnibus set (GSE36681) showed that miR-205 expression was upregulated in human NSCLC tissues (Figure 5B). Interestingly, the ratio of miR-205 level (Tumor/Normal; T/N) was inversely correlated with the ratio of *SMAD4* mRNA levels (T/N) in 52 paired tissues (*P* = 0.0065; Figure 5C). Further analysis showed that 42 NSCLC tissues had high miR-205 level while 37 tissues (88.1%) had low expression of *SMAD4* mRNA. Eight NSCLC tissues had low miR-205 level, while three tissues (37.5%) had high expression of *SMAD4* mRNA (Figure 5D).

Furthermore, to determine the function of miR-205 in NSCLC, taking into account that miR-205 is downregulated significantly in NSCLC cell lines [27, 28], we overexpressed miR-205 in NSCLC cells using miR-205 mimics and then evaluated the effect of miR-205 on cell growth (Figure 6A). CCK-8 assays showed that NSCLC cells overexpressing miR-205 had significantly higher proliferation abilities compared with control cells (Figure 6B). The results were confirmed by a clonogenic assay in A549 cells (Figure 6C), suggesting that miR-205 promotes NSCLC cell proliferation.

### Overexpression of miR-205 accelerates the cell cycle in NSCLC cells

**Figure 7:**
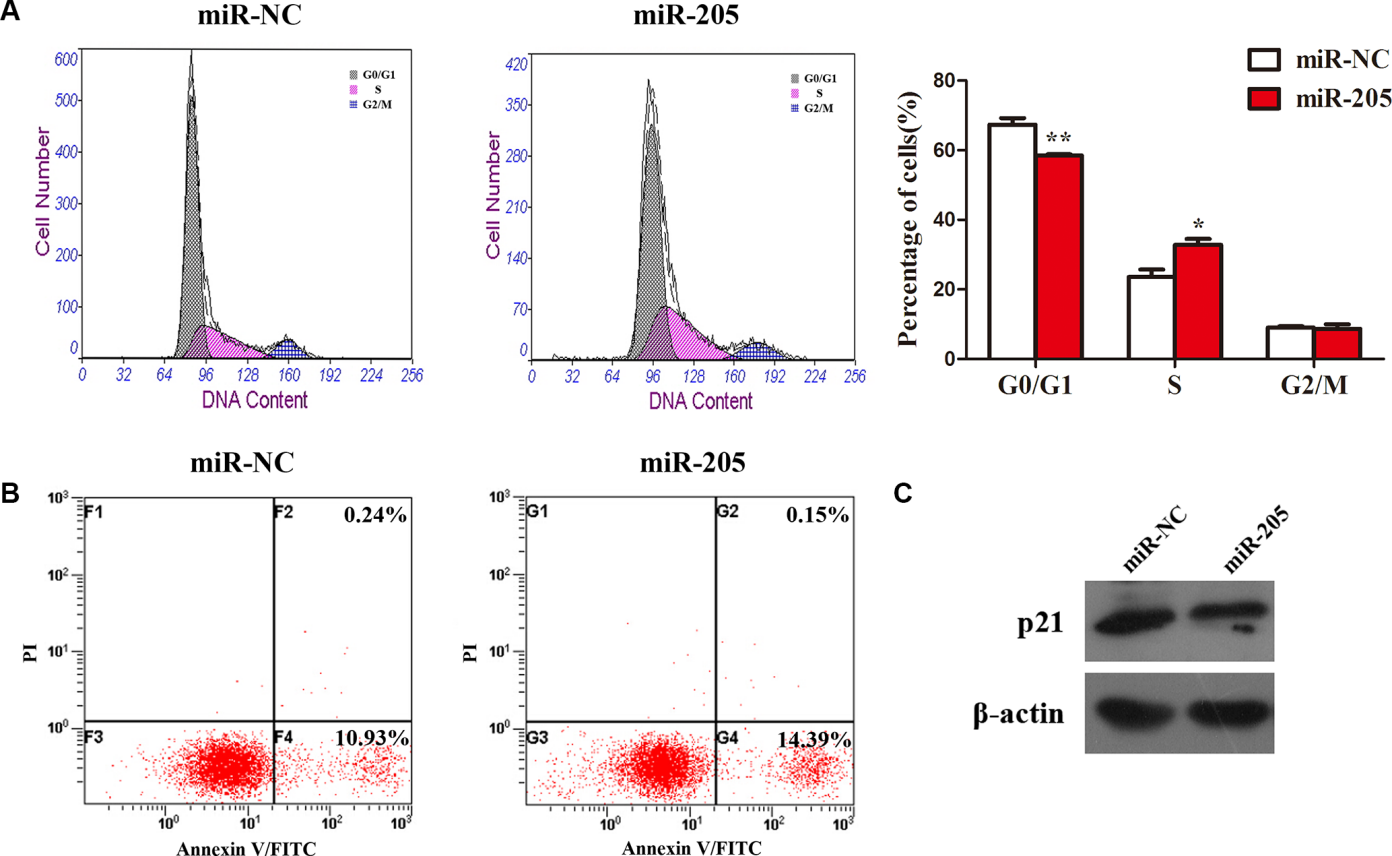
Overexpression of an miR-205 mimic had no effect on cell apoptosis, but promoted the cell cycle in NSCLC cells (**A**) Flow cytometry cell cycle analysis of A549 cells with miR-205 mimics or NC. Cells were harvested at 72 h post-transfection and stained with propidium iodide. Shown in the inset of each panel are the percentages of cell cycle phases, in which values represent the mean ± SD of three measurements. (**B**) Flow cytometry apoptosis assay of A549 cells with miR-205 mimics or NC. Cells were harvested at 72 h post-transfection and stained with Annexin V/FITC and propidium iodide (PI). (**C**) Expression of p21 (an inhibitor of cell proliferation) was analyzed by western blotting. Densitometry values for each protein were normalized to that of β-actin. *P < 0.05; **P < 0.01; ***P < 0.001.

To determine how miR-205 promotes cell proliferation in NSCLC cells, we examined cell apoptosis and the distribution of cell cycle phases in A549 cells overexpressing miR-205 and NC. We found that overexpression of miR-205 mimics had no effect on cell apoptosis (Figure 7B), whereas ectopic expression of miR-205 led to a significant reduction in the number of cells in the G1 phase (Figure 7A). In addition, the expression of p21 was increased in cell ectopically expressing miR-205 (Figure 7C). Collectively, the results suggested that miR-205 promotes cell proliferation by accelerating NSCLC cell cycle.

### MiR-205 promotes tumor growth of lung carcinoma xenografts in nude mice

**Figure 8:**
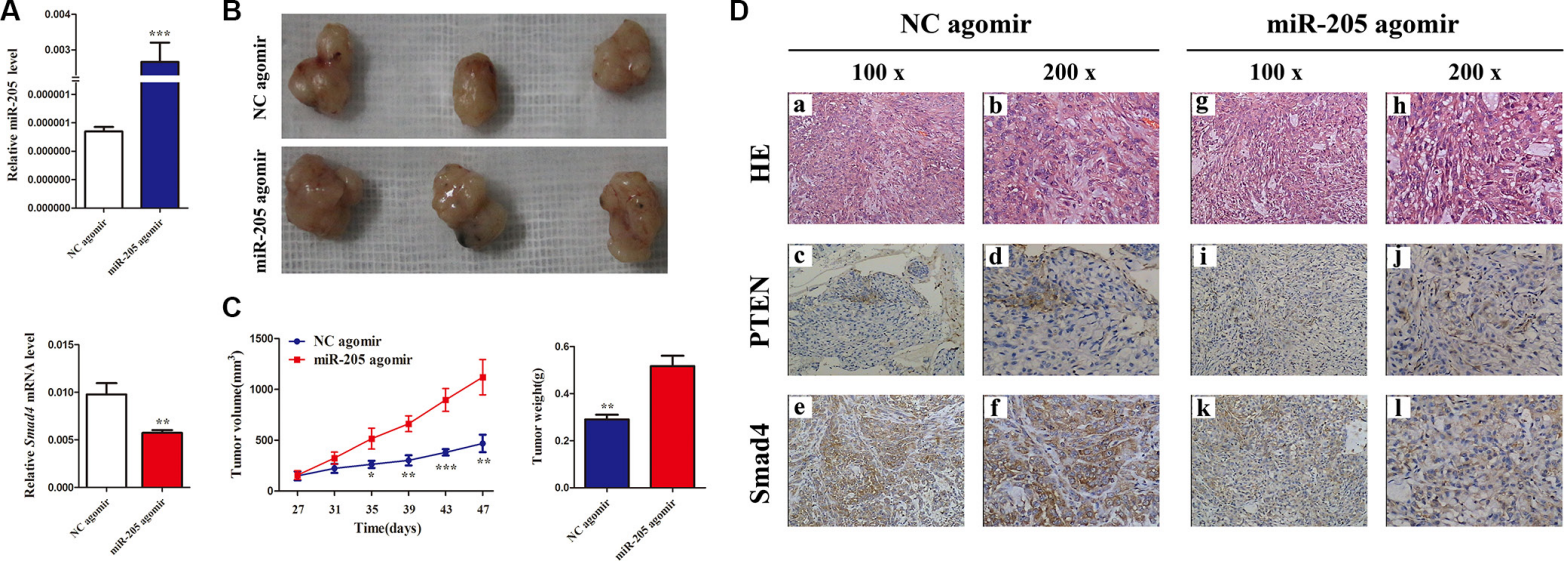
Overexpressing *SMAD4* in NSCLC cells promotes tumor growth *in vivo* (**A**) At the experimental endpoint, tumors were dissected and photographed as indicated. (**B**) Tumor growth curves in mice (*n* = 3/group) inoculated with the indicated cells at the indicated days; each tumor formed by the indicated cells was weighed. (**C**) Hematoxylin and eosin (H and E) staining confirmed tumor cells in slices of the indicated tumor sections. Immunohistochemical staining for SMAD4 was quantified using its staining intensity.

We next sought to clarify the cellular mechanisms underlying miR-205-mediated tumor suppression. Firstly, control A549 cells and the corresponding cells that overexpress *SMAD4* stably were inoculated into BALB/C athymic mice. As shown in Figure 8A and 8B, tumors formed in mice injected with *SMAD4*-overexpressing cells were larger and heavier compared with those of the control. Subsequently, the resected tissues from the xenograft tumors were analyzed to verify SMAD4 expression using IHC (Figure 8C).

**Figure 9:**
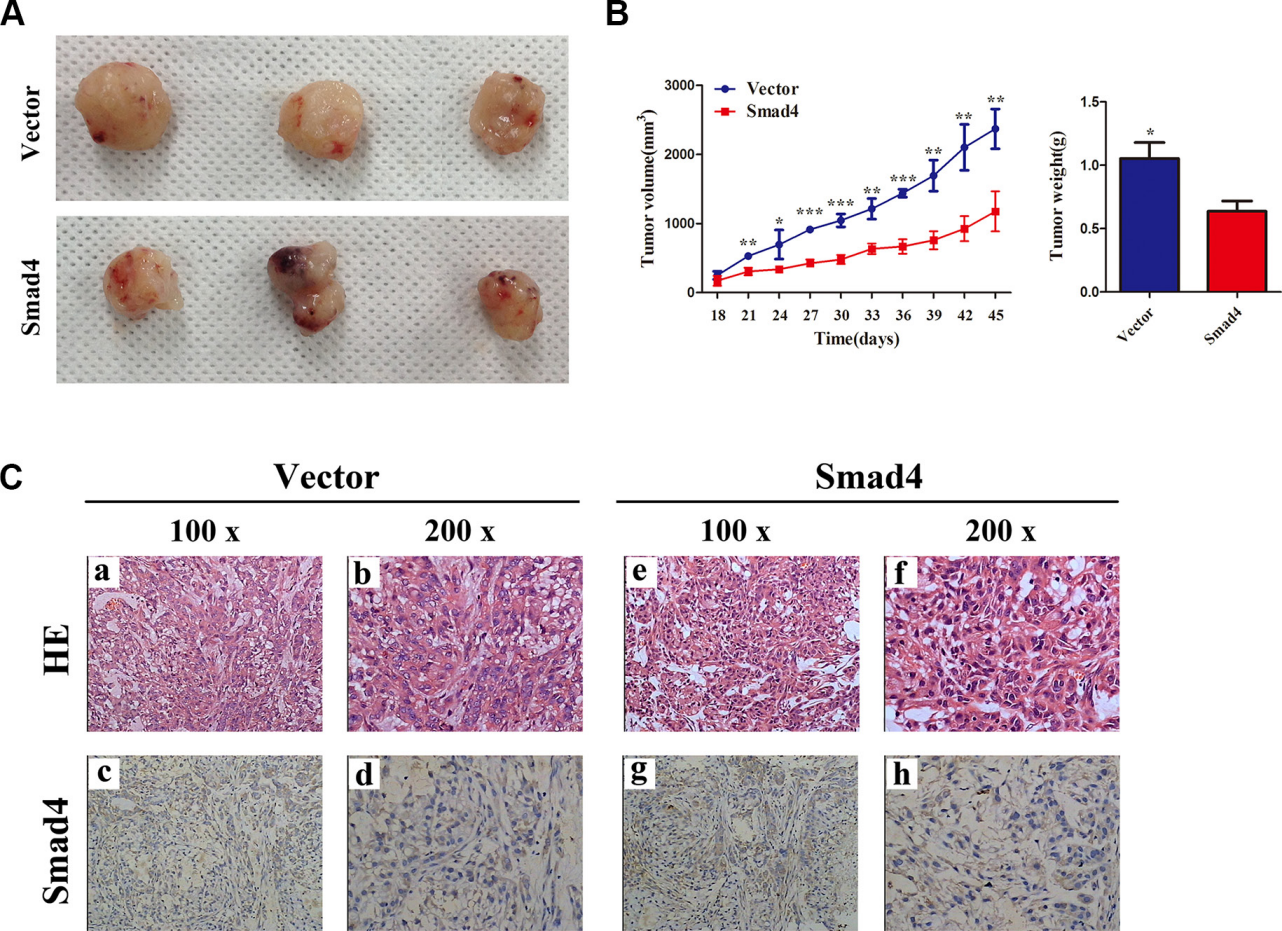
Overexpressing miR-205 in lung carcinoma xenografts promotes tumor growth *in vivo* (**A**) QRT-PCR analysis of miR-205 levels and *SMAD4* mRNA expression in excised tumors transfected with miR-205 agomir and NC agomir; U6 and β-actin were used as internal controls, respectively. (**B**) At the experimental endpoint, tumors were dissected and photographed as indicated. (**C**) Tumor growth of miR-205 agomir and NC agomir-treated A549 xenografts in nude mice (*n* = 3). The graph show the tumor growth curves at sacrifice with respect to the first measurements, using the administration of 1 nmol miR-205 agomir or NC agomir per mouse every 4 days for seven times; the arrows indicate the weight of the excised tumors (mean ± SD, *n* = 3). (**D**) Hematoxylin and eosin (H and E) staining confirmed tumor cells in slices of the indicated tumor sections. Immunohistochemical staining for SMAD4 and PTEN was quantified using the staining intensity.

Considering the important functions of miR-205 and SMAD4 in NSCLC, the potential therapeutic use of miR-205 attracted our attention. MiR-205 is significantly downregulated in NSCLC cell lines; therefore, an miR-205 agomir was prepared for replacement therapy. As shown in Figure 9A, the miR-205 agomir increased the expression of miR-205 significantly and decreased the expression of *SMAD4*. Tumors treated with the miR-205 agomir were larger and heavier compared with those from the control (Figure 9B and 9C). In addition, the resected tissues from the agomir-treated xenograft tumors were analyzed to verify PTEN and SMAD4 expression using IHC: consistent with the above observations, significant loss of SMAD4 expression was shown in miR-205 agomir group comparied with the NC group (Figure 9D). All these data indicated that re-expression of miR-205 could promote lung cancer cell growth *in vivo* by inhibiting the expression *SMAD4*.

## DISCUSSION

Although there has been an incremental improvement in the survival rate over the last several decades, advances in survival seen in other common malignancies have not been realized in lung cancer, which is still the leading cause of cancer mortality worldwide, including China [26]. The current 5-year survival rate for lung cancer is a discouraging 15%.

The molecular genetic alterations, including genetic and epigenetic changes, could occur before morphological changes can be detected by a cytological test [27–30]. Recently, accumulated studies have profiled miRNA expression directly in NSCLC, and particular group of miRNAs were identified that either characterize the neoplastic tissues or identify patients with poor prognosis [10, 31–33].

Each miRNA has the potential to target hundreds of genes that harbor sequences in their 3′-UTRs that are complementary to the seed region of the miRNA [3, 4]. For lung cancer, it has been shown recently that the expression of miRNAs of the let-7 family were frequently reduced both *in vivo* and *in vitro*, and that reduced let-7 expression was significantly associated with shortened postoperative survival [33]. MiR-205 is localized at 1q32.2, which is a lung cancer-associated genomic amplification region [10, 32]. The above studies suggested that miR-205 acts either as an oncogene or tumor suppressor gene, depending on the cellular environment. Indeed, its tumor promoting and suppressing roles have been demonstrated in different cancer cell lines. For example, miR-205 suppresses cell migration and invasion via the epithelial-to-mesenchymal transition in human prostate and breast cancer cells [13, 34], In support of its oncogenic function, miR-205 was binds to *PETN* and *PHLPP2* to modulate PI3K/AKT signaling and promote cell proliferation in NSCLC [12]. These findings indicated that the stimulation or inhibition of miR-205 expression, as well as its biological functions, might be tissue or cancer-type dependent. Thus, it remains important to understand thoroughly the molecular mechanisms underlying the differential biological effects and targets of miR-205 in NSCLC and other cancer types. Given the complexity of its function, it would be interesting to investigate the correlation miR-205 expression with the activity of the TGF-beta signaling pathway in NSCLC. In the present study, miR-205 expression was increased while *SMAD4* was decreased in NSCLC, such that that the ratio of miR-205 level (T/N) was inversely correlated with that of the *SMAD4* mRNA level (T/N) in 52 paired tissues (*P* = 0.0065).

SMAD4 is a member of the Smad family that functions in the transforming growth factor beta (TGF-β) signaling pathway. SMAD4 activation under different conditions could lead to apoptosis or growth arrest in the G1 phase of the cell cycle, which are responses associated mainly with tumor development [35]. In addition, patients with high rates of lymph node involvement show SMAD4-negative expression. SMAD4 inactivation in colon cancer patients was more likely to be observed in older patients and in those with a later tumor stage. However, in pancreatic cancer, there was no obvious relationship observed between SMAD4 levels and clinical parameters. These results suggested that SMAD4′s biology or mechanism of action is different for different types of tumor. Therefore, further studies including different types of cancer are needed to judge the association of clinical parameters with SMAD4 levels, and to reveal the associated mechanisms. A previous study showed that the use of a *DPC4* siRNA in A549 cells also decreased VEGF protein and mRNA expression [17]. In our study, cell growth was significantly increased in cells transfected with si-Smad4 compared with the control cells. By contrast, the growth of cells overexpressing *SMAD4* was significantly suppressed compared with the control cells. Furthermore, to validate these observations, we also transfected miR-205 mimics into NSCLC cells and injected miR-205 agomirs into implanted tumors: similar results were observed. Taken together, we believe thatmiR-205 promotes cell proliferation by repressing the expression of *SMAD4* directly in NSCLC.

In conclusion, our study shows that miR-205 suppresses the expression of tumor suppressor gene *SMAD4* promotes NSCLC cells growth *in vitro* and *in vivo*. Our findings also highlighted the therapeutic potential of miR-205 in the treatment of NSCLC and supported the development of effective therapeutic strategies that target miR-205 (or its targets, such as *SMAD4*) via a genetic or pharmacological approach.

## MATERIALS AND METHODS

### Tissue samples

Fifty-two paired NSCLC tissues and adjacent noncancerous lung tissues from the cancer edge were obtained after informed consent from patients in the First Affiliated Hospital of Soochow University between 2009 and 2013. Histological and pathological diagnosis of patients with NSCLC was performed according to the Revised International System for Staging Lung Cancer. The NSCLC patients had received neither chemotherapy nor radiotherapy before tissue sampling. Tissue samples were snap-frozen and stored at −80°C. This study was approved by the Ethics Committee of the First Affiliated Hospital of Soochow University.

### Cell culture

Human lung carcinoma cell lines (A549, H1299, H1650, SPC-A1, H460, 95C, 95D, H226, H520 and SK-MES-1) were purchased from the Cell Bank of the Chinese Academy of Sciences (Shanghai, China), and human bronchial epithelial (HBE) cells were from Bogoo Biotechnology (Shanghai, China). Cells were seeded and grown in RPMI 1640 medium (HyClone, South Logan, UT, USA) with 10% heat-inactivated fetal bovine serum (Gibco, Carlsbad, CA, USA), L-glutamine and antibiotics (Invitrogen, Carlsbad, CA, USA) in a humidified incubator containing 5% CO_2_ at 37°C.

### RNA extraction, cDNA synthesis and qRT-PCR

Total RNA of cells and tissues was extracted by adding 1.0 ml RNAiso Plus (Takara, Osaka, Japan), according to the manufacturer′s protocol. The concentration of RNA was measured using a NanoDrop 2000 (Thermo Fisher Scientific, Waltham, MA, USA). Synthesis of cDNA was carried out with reverse transcriptase M-MLV (Takara). The Primers for reverse transcription and amplification of miR-205 and U6 were designed and synthesized by Guangzhou RiboBio Company (Guangzhou, China). The primer sequences for qRT-PCR of *SMAD4* and β-actin (*ACTB*) were as follows: *Smad4*, Forward: 5′-CAGCCATC-GTTGTCCACT-3′, Reverse: 5′-GCTGGGGTGCTGTATGTC-3′, *ACTB*, Forward: 5′-CACAGAGCCTCGCCTTT GCC-3′, Reverse: 5′-ACCCATGCCCACCATCACG-3′, qRT-PCR was performed using SYBR Premix ExTaq^™^ (Takara), according to the manufacturer's instructions, on an ABI Step One Plus Real-Time PCR system (Applied Biosystems, Foster City, CA, USA). The PCR program comprised 50°C for 2 min; 95°C for 10 min; followed by 45 cycles of 95°C for 15 sec and 60°C for 1 min. mRNA expression values of *Smad4* and miR-205 were normalized to the internal controls *ACTB* and U6, respectively. Relative expression was calculated using the cycle threshold (Ct) method.

### Western blotting

Cells were grown to 80%~90% confluence and then lysed in a RIPA buffer (Cell Signaling Technology, Danvers, MA, USA) with protease inhibitor and phosphatase inhibitor cocktail (Sigma-Aldrich, St. Louis, MO, USA) and centrifuged. Total cell lysates were separated by 10% SDS-PAGE, transferred to nitrocellulose membranes (Millipore, Billerica, MA, USA), blocked with 5% BSA in TBST buffer with 0.1% Tween-20 for 1 h at room temperature and incubated with primary antibodies overnight at 4°C. After washing four times with TBST, the membranes were incubated with the corresponding HRP-conjugated secondary antibodies for 2 h at room temperature. Detection was performed using and ECL kit (Pierce, Rockford, IL, USA). The band density was quantified using Quantity One 4.6 software. All antibodies used for western blotting, including anti-SMAD4, anti-p21, anti-β-actin, anti-mouse and anti-goat secondary antibodies were from Cell Signaling Technology.

### Construction of luciferase reporter plasmids, transient transfection, and luciferase assay

We used the psiCHECK2 dual luciferase vector (Promega, Madison, WI, USA) to construct a plasmid containing the *SMAD4* 3′-UTR fused to the 3′ end of a luciferase reporter. Briefly, a 215-bp fragment containing predicted miR-205 target site (positions 262–269) was chosen for the luciferase assay. The wild-type and mutated fragments were synthesized directly (Genewiz, Suzhou, China), and then subcloned into the psiCHECK2 vector to generate psiCHECK2-SMAD4-3′-UTR wild-type and a psiCHECK2-Smad4-3′-UTR-mutant. Subsequently, A549 cells were plated in a 24-well plate and cotransfected with the wild-type or mutated plasmid, control pRL-TK plasmid and with either miR-205 mimics or miR-negative control (NC) using Lipofectamine 2000 (Life Technologies). After 48 h, cells were collected, and luciferase activities were measured using Dual-Luciferase Reporter Assay Kit (Promega). Each experiment was performed in triplicate.

### MiR-205 precursor, plasmid, siRNA and cell transfection

MiR-205 mimics and matched NC were from RiboBio Co., Ltd (Guangzhou, China). The control and SMAD4 siRNAs were prepared as previously described [36]. The target sequences of the siRNA was: 5′-GTACTTCATACCATGCCGA-3′; Cell transfections were performed using Lipofectamine 2000 (Invitrogen, Carlsbad, CA, USA), according to the manufacturer's instruction. After 72 h of transfection, the cells were collected for further experiments.

### Cell proliferation analysis

The Cell Counting Kit-8 assay kit (CCK-8, Boster, Wuhan, China) was used to assess cell proliferation. A549 cells and stable cell lines overexpressing SMAD4 were plated in 6-well plates under normal culture conditions, and then transfected with si-SMAD4 and NC or miR-205 mimics. After 48 h, the cells were digested and seeded in 96-well plates (2 × 10^3^ cells/well). We also detected cell proliferation using a clonogenic assay. Briefly, cells transfected with miR-205 mimics and si-SMAD4 or NC were diluted in complete culture medium and 200 cells were reseeded in a 60 mm plate. After incubation for 14–20 days, depending on cell growth rate, foci formed by least 50 cells were stained with Giemsa and counted. Cell viability was measured according to manufacturer's instructions at several time points (24, 48 and 72 h). Each experiment was performed in triplicate.

### Cell cycle analysis

According to the instructions of the Cell Cycle Analysis Kit (Beyotime, Shanghai, China), cells were cultured in 6-well plates, transfected with miR-NC, miR-205, Si-NC or Si-SMAD4 for 72 h. The cells were then collected, washed with cold phosphate-buffered saline (PBS), fixed in 70% ethanol at 4°C for 24 h, washed with cold PBS again and stained in a Propidium Iodide (PI)/RNase A mixture. After being kept in the darker at 37°C for 30 min, the cells were analyzed using a fluorescence-activated cell sorting (FACS) Caliber system (Beckman Coulter, Brea, CA, USA).

### Cell apoptosis analysis

Cells were transfected with miR-NC, miR-205, Si-NC or Si-SMAD4. After 72 h, cells were harvested, washed, resuspended in the binding buffer containing Annexin V/FITC and PI (Beyotime). The stained cells were then detected using a fluorescence-activated cell sorting (FACS) Caliber system (Beckman Coulter).

### Generation of stable cell lines overexpressing SMAD4

To generate A549 cells in which SMAD4 can stably overexpressed, we subcloned the coding sequence of *SMAD4* into a pLVX-IRES-Neo vector using endonucleases EcoRI and BamHI, for expression via a Lenti-X lentiviral expression system (Clontech, Mountain View, CA, USA). The SMAD4 expression construct was co-transfected with packaging plasmids into human embryonic kidney 293 T cells using Lipofectamine 2000 (Invitrogen). The empty vector served as a negative control. Human embryonic kidney 293 T cells were cultured in Dulbecco's modified Eagle's medium with 10% fetal bovine serum at 37°C in a humidified 5% CO_2_ incubator for 48 h. After the incubation, the packaged lentiviruses were collected and used to infect A549 cells. After 2 days, stable cells were selected with 400 μg/ml of G418 (Amresco, Solon, OH, USA). The coding sequence region of *SMAD4* was amplified using the following primers: forward, 5′-CAGCCATC-GTTGTCCACT-3′; reverse: 5′-GCTGGGGTGCTGTATGTC-3′.

### Animal experiments and immunocytochemical staining

BALB/c athymic nude mice (female, 4–6-weeks old and 16–20 g) were purchased from the Experimental Animal Center of Soochow University and bred under pathogen-free conditions. All animal experiments were carried out in accordance with the Guide for the Care and Use of Experimental Animal Center of Soochow University. To establish a lung carcinoma xenograft model, A549 cells and A549 cells in which SMAD4 can be stably overexpressed were suspended in 100 ml of PBS and inoculated subcutaneously into the flanks of nude mice. After 8–10 days, the transplanted nude mice were randomly divided into four groups (*n* = 8 each). miR-205 agomir and NC agomir (RiboBio Co, Ltd) were injected directly into the implanted tumor at a dose of 1 nmol (in 20 ml PBS) per mouse every 4 days, seven times. These chemically stabilized miRNAs are thought to have markedly improved pharmacological properties [37]. Tumor volume (*V*) was monitored by measuring the length *(L*) and width (*W*) using Vernier calipers and calculated using the formula *V* = (*L* × W2) × 0.5.

Immunohistochemical (IHC) analyses of tissues were conducted as described in our previous study [38]. In brief, sections were deparaffinized, the antigen was retrieved in a Decloaking Chamber™ (Biocare Medical, Concord, CA, USA) in the presence of 10 mM citrate buffer (pH 6.0). After blocking in goat serum, the sections were incubated with 1:100 diluted PTEN and SMAD4-specific monoclonal antibodies (Santa Cruz Biotechnology, Santa Cruz, CA, USA) overnight at 4°C, followed by incubation with biotinylated secondary antibody, developed using a diaminobenzidine (DAB) Kit (BD Bioscience, San Jose, CA, USA) and counterstained with hematoxylin.

### Statistical analysis

Differences in miR-205 and SMAD4 expression between NSCLC tissues (T) and adjacent noncancerous lung tissues (N) were analyzed using a paired *t* test (two-tailed). For cell lines, differences between two groups were assessed using an unpaired *t* test (two-tailed). Comparisons between clinicopathological characteristics and expression levels of mRNA in NSCLC samples were performed using nonparametric tests (unpaired *t* test for two groups; Kruskall–Wallis test for three or more groups). The data are presented as mean ± standard error (SE). Statistical differences were considered significant at *P* < 0.05. All statistical analyses were performed using GraphPad Prism 5.02 (GraphPad, San Diego, CA, USA) and SPSS 16.0 software (SPSS, Chicago, IL, USA).

## References

[1] Jemal A, Bray F, Center MM, Ferlay J, Ward E, Forman D (2011). Global cancer statistics. CA Cancer J Clin.

[2] Wang T, Nelson RA, Bogardus A, Grannis FW (2010). Five-year lung cancer survival: which advanced stage nonsmall cell lung cancer patients attain long-term survival?. Cancer.

[3] Guo H, Ingolia NT, Weissman JS, Bartel DP (2010). Mammalian microRNAs predominantly act to decrease target mRNA levels. Nature.

[4] Wilson RC, Doudna JA (2013). Molecular mechanisms of RNA interference. Annu Rev Biophys.

[5] Lewis BP, Burge CB, Bartel DP (2005). Conserved seed pairing, often flanked by adenosines, indicates that thousands of human genes are microRNA targets. Cell.

[6] Bueno MJ, Perez de Castro I, Malumbres M (2008). Control of cell proliferation pathways by microRNAs. Cell Cycle.

[7] Lee CT, Risom T, Strauss WM (2006). MicroRNAs in mammalian development. Birth Defects Res C Embryo Today.

[8] Jovanovic M, Hengartner MO (2006). miRNAs and apoptosis: RNAs to die for. Oncogene.

[9] Lu J, Getz G, Miska EA, Alvarez-Saavedra E, Lamb J, Peck D, Sweet-Cordero A, Ebert BL, Mak RH, Ferrando AA, Downing JR, Jacks T, Horvitz HR (2005). MicroRNA expression profiles classify human cancers. Nature.

[10] Lebanony D, Benjamin H, Gilad S, Ezagouri M, Dov A, Ashkenazi K, Gefen N, Izraeli S, Rechavi G, Pass H, Nonaka D, Li J, Spector Y (2009). Diagnostic assay based on hsa-miR-205 expression distinguishes squamous from nonsquamous non-small-cell lung carcinoma. J Clin Oncol.

[11] Markou A, Tsaroucha EG, Kaklamanis L, Fotinou M, Georgoulias V, Lianidou ES (2008). Prognostic value of mature microRNA-21 and microRNA-205 overexpression in non-small cell lung cancer by quantitative real-time RT-PCR. Clin Chem.

[12] Cai J, Fang L, Huang Y, Li R, Yuan J, Yang Y, Zhu X, Chen B, Wu J, Li M (2013). miR-205 targets PTEN and PHLPP2 to augment AKT signaling and drive malignant phenotypes in non-small cell lung cancer. Cancer Res.

[13] Gregory PA, Bert AG, Paterson EL, Barry SC, Tsykin A, Farshid G, Vadas MA, Khew-Goodall Y, Goodall GJ (2008). The miR-200 family and miR-205 regulate epithelial to mesenchymal transition by targeting ZEB1 and SIP1. Nat Cell Biol.

[14] Chao CH, Chang CC, Wu MJ, Ko HW, Wang D, Hung MC, Yang JY, Chang CJ (2014). MicroRNA-205 signaling regulates mammary stem cell fate and tumorigenesis. J Clin Invest.

[15] Zhou S, Buckhaults P, Zawel L, Bunz F, Riggins G, Dai JL, Kern SE, Kinzler KW, Vogelstein B (1998). Targeted deletion of Smad4 shows it is required for transforming growth factor beta and activin signaling in colorectal cancer cells. Proc Natl Acad Sci U S A.

[16] Lagna G, Hata A, Hemmati-Brivanlou A, Massague J (1996). Partnership between DPC4 and SMAD proteins in TGF-beta signalling pathways. Nature.

[17] Ke Z, Zhang X, Ma L, Wang L (2008). Expression of DPC4/Smad4 in non-small-cell lung carcinoma and its relationship with angiogenesis. Neoplasma.

[18] Chen H, Wang JW, Liu LX, Yan JD, Ren SH, Li Y, Lu Z (2015). Expression and significance of transforming growth factor-beta receptor type II and DPC4/Smad4 in non-small cell lung cancer. Exp Ther Med.

[19] Stuelten CH, Buck MB, Dippon J, Roberts AB, Fritz P, Knabbe C (2006). Smad4-expression is decreased in breast cancer tissues: a retrospective study. BMC Cancer.

[20] Ding Z, Wu CJ, Chu GC, Xiao Y, Ho D, Zhang J, Perry SR, Labrot ES, Wu X, Lis R, Hoshida Y, Hiller D, Hu B (2011). SMAD4-dependent barrier constrains prostate cancer growth and metastatic progression. Nature.

[21] Meulmeester E, Ten Dijke P (2011). The dynamic roles of TGF-beta in cancer. J Pathol.

[22] Zhu J, Zeng Y, Xu C, Qin H, Lei Z, Shen D, Liu Z, Huang JA (2015). Expression profile analysis of microRNAs and downregulated miR-486-5p and miR-30a-5p in non-small cell lung cancer. Oncology Reports.

[23] Huang W, Jin Y, Yuan Y, Bai C, Wu Y, Zhu H, Lu S (2014). Validation and target gene screening of hsa-miR-205 in lung squamous cell carcinoma. Chin Med J.

[24] Du L, Schageman JJ, Irnov Girard L, Hammond SM, AF MinnaJD Gazdar, Pertsemlidis A (2010). MicroRNA expression distinguishes SCLC from NSCLC lung tumor cells and suggests a possible pathological relationship between SCLCs and NSCLCs. J Exp Clin Cancer Res.

[25] Larzabal L, de Aberasturi AL, Redrado M, Rueda P, Rodriguez MJ, Bodegas ME, Montuenga LM, Calvo A (2014). TMPRSS4 regulates levels of integrin α5 in NSCLC through miR-205 activity to promote metastasis. Br J Cancer.

[26] Chen W, Zheng R, Baade PD, Zhang S, Zeng H, Bray F, Jemal A, Yu XQ, He J (2016). Cancer statistics in China. CA Cancer J Clin.

[27] Belinsky SA, Liechty KC, Gentry FD, Wolf HJ, Rogers J, Vu K, Haney J, Kennedy TC, Hirsch FR, Miller Y, Franklin WA, Herman JG, Baylin SB (2006). Promoter hypermethylation of multiple genes in sputum precedes lung cancer incidence in a high-risk cohort. Cancer Res.

[28] Jiang F, Todd NW, Qiu Q, Liu Z, Katz RL, Stass SA (2009). Combined genetic analysis of sputum and computed tomography for noninvasive diagnosis of non-small-cell lung cancer. Lung Cancer.

[29] Liu Z, Zhao J, Chen XF, Li W, Liu R, Lei Z, Liu X, PengX Xu K, Chen J, Liu H, Zhou QH, Zhang HT (2008). CpG island methylator phenotype involving tumor suppressor genes located on chromosome 3p in non-small cell lung cancer. Lung Cancer.

[30] Liu Z, Li W, Lei Z, Zhao J, Chen XF, Liu R, Peng X, Wu ZH, Chen J, Liu H, Zhou QH, Zhang HT (2010). CpG island methylator phenotype involving chromosome 3p confers an increased risk of non-small cell lung cancer. J Thorac Oncol.

[31] Yu SL, Chen HY, Chang GC, Chen CY, Chen HW, Singh S, Cheng CL, Yu CJ, Lee YC, Chen HS, Su TJ, Chiang CC, Li HN (2008). MicroRNA signature predicts survival and relapse in lung cancer. Cancer Cell.

[32] Yanaihara N, Caplen N, Bowman E, Seike M, Kumamoto K, Yi M, Stephens RM, Okamoto A, Yokota J, Tanaka T, Calin GA, Liu CG, Croce CM (2006). Unique microRNA molecular profiles in lung cancer diagnosis and prognosis. Cancer Cell.

[33] Takamizawa J, Konishi H, Yanagisawa K, Tomida S, Osada H, Endoh H, Harano T, Yatabe Y, Nagino M, Nimura Y, Mitsudomi T, Takahashi T (2004). Reduced expression of the let-7 microRNAs in human lung cancers in association with shortened postoperative survival. Cancer Res.

[34] Gandellini P, Folini M, Longoni N, Pennati M, Binda M, Colecchia M, Salvioni R, Supino R, Moretti R, Limonta P, Valdagni R, Daidone MG, Zaffaroni N (2009). miR-205 Exerts tumor-suppressive functions in human prostate through down-regulation of protein kinase Cepsilon. Cancer Res.

[35] Dai JL, Bansal RK, Kern SE (1999). G1 cell cycle arrest and apoptosis induction by nuclear Smad4/Dpc4: phenotypes reversed by a tumorigenic mutation. Proc Natl Acad Sci U S A.

[36] Yang H, Wang L, Zhao J, Chen Y, Lei Z, Liu X, Xia W, Guo L, Zhang HT (2015). TGF-β-activated SMAD3/4 complex transcriptionally upregulates N-cadherin expression in non-small cell lung cancer. Lung Cancer.

[37] He XX, Chang Y, Meng FY, Wang MY, Xie QH, Tang F, Li PY, Song YH, Lin JS (2012). MicroRNA-375 targets AEG-1 in hepatocellular carcinoma and suppresses liver cancer cell growth *in vitro* and *in vivo*. Oncogene.

[38] Zhou P, Erfani S, Liu Z, Jia C, Chen Y, Xu B, Deng X, Alfáro JE, Chen L, Napier D, Lu M, Huang JA, Liu C (2015). CD151-α3β1 integrin complexes are prognostic markers of glioblastoma and cooperate with EGFR to drive tumor cell motility and invasion. Oncotarget.

